# Adverse drug reactions to antiretroviral therapy (ARVs): incidence, type and risk factors in Nigeria

**DOI:** 10.1186/1472-6904-12-7

**Published:** 2012-02-27

**Authors:** George I Eluwa, Titilope Badru, Kesiena J Akpoigbe

**Affiliations:** 1 Department of Operations Research, HIV/AIDS Program. Population Council, Nigeria. Plot 759, Cadastral Zone AO, Off Constitution Avenue, Central Business District, Abuja, Nigeria; 2 Department of Health Policy and Management, Diadem Consults Ltd, Abuja, Nigeria; 3 Society for Family Health, Abuja, Nigeria

**Keywords:** Adverse drug reactions, ADR, Antiretroviral, Zidovudine, Stavudine, Tenofovir, HIV/AIDS, Nigeria, Incidence, Risk factors

## Abstract

**Background:**

Data on adverse drug reactions (ADRs) related to antiretroviral (ARV) use in public health practice are few indicating the need for ART safety surveillance in clinical care.

**Objectives:**

To evaluate the incidence, type and risk factors associated with adverse drug reactions (ADRs) among patients on antiretroviral drugs (ARV).

**Methods:**

Patients initiated on ARVs between May 2006 and May 2009 were evaluated in a retrospective cohort analysis in three health facilities in Nigeria. Regimens prescribed include nucleoside backbone of zidovudine (AZT)/lamivudine (3TC), stavudine (d4T)/3TC, or tenofovir (TDF)/3TC in combination with either nevirapine (NVP) or efavirenz (EFV). Generalized Estimating Equation (GEE) model was used to identify risk factors associated with occurrence of ADR.

**Results:**

2650 patients were followed-up for 2456 person-years and reported 114 ADRs (incidence rate = 4.6/100 person-years).There were more females 1706(64%) and 73(64%) of the ADRs were reported by women. Overall, 61(54%) of ADRs were reported by patients on AZT with 54(47%) of these occurring in patients on AZT/NVP. The commonest ADRs reported were pain 25(30%) and skinrash 10(18%). Most ADRs were grade 1(39%) with only 1% being life threatening (grade 4). Adjusted GEE analysis showed that ADR was less likely to occur in patients on longer duration of ART compared to the first six months on treatment; 6-12 months AOR 0.38(95% CI:0.16-0.91) and 12-24 months AOR 0.34(95% CI:0.16-0.73) respectively. Compared to patients on TDF, ADR was less likely to occur in patients on d4T and AZT AOR 0.18(95% CI 0.05-0.64) and AOR 0.24(95% CI:0.7-0.9) respectively. Age, gender and CD4 count were not significantly associated with ADRs.

**Conclusion:**

ADRs are more likely to occur within the first six months on treatment. Close monitoring within this period is required to prevent occurrence of severe ADR and improve ART adherence. Further research on the tolerability of tenofovir in this environment is recommended.

## Background

The concerted efforts of developed nations and international organizations have significantly reduced the impact of the HIV epidemic in developing countries by providing the means to scale up care and treatment. Millions of eligible HIV infected patients have access to life prolonging antiretroviral (ARV) drugs. This has led to appreciable decrease in HIV related morbidity and mortality [1-4]. Like most chronically administered drugs, ARVs have documented toxicities and adverse effects. ADRs range from mild to life threatening with short and long term effects, however little is known about the adverse drug reactions (ADR) of ARVs in many HIV programs in the public health sector of developing countries [2]. The spectrum of adverse effects associated with ARVs may vary between developed and developing countries [5]. Variance in psychological and socioeconomic support of HIV positive patients in the public health sector of developing countries coupled with co-morbidities make monitoring ADRs to antiretroviral a necessity. Studies on the incidence of ADR from developing and developed countries have reported incidence of ADR among patients on ARVs to range between 11%-35.9% [6,7] with incidence as high as 54% [8] in the presence of opportunistic infection. Incidence of severe ADR has been reported to be as high as 10% [6] with a study observing an incidence rate of 8 per 100 person years [4]. The long term effects of ARTs are largely unknown but ongoing research provides insights into some ADRs of ARV [9]. These include peripheral neuropathy and lipodystrophy associated with stavudine,[3,4] anaemia associated with zidovudine [10,11] and nevirapine based hepatotoxicity and rash [12-22]. Incidence of hepatotoxicity was observed to be 16% and 8% for patients on NVP and EFV respectively [23] while incidence of anaemia ranged from 3- 12% among patients on zidovudine in developing countries including Nigeria [5].

There is substantial evidence linking treatment success to adherence to ARVs [4,15]. However adherence to treatment is closely linked to adverse drug reactions [4,9,15]. It is thus imperative that clinicians clearly understand ADRs, readily recognize them in patients and manage them effectively. Most studies on ADRs are clinical trials and represent a select group of cohort; however studies of large cohorts of unselected patients are more suited [4] to inform on the situation of ADRs in actual clinical practice of the public health sector.

Nigerian operates a universal health care system which supports the provision of primary, secondary and tertiary levels of health services [24]. Primary health care is funded by the local government, while secondary and tertiary level care is funded by the state and federal government respectively. Delivery of healthcare is decentralized to the state level, leading to much variation in resources and funding. In 2004, Nigeria received over $400 million dollars in funding to scale up ART and part of this fund was implemented by Family Health International under the Global HIV/AIDS Initiative Nigeria (GHAIN) project. This resulted in the influx of ART into the country on a large scale.

Admission into the GHAIN supported ART program represents the typical approach to ART care and treatment in public health setting. Typically it starts with the HIV counselling and testing and determination of the patients HIV status using rapid test kits. The eligibility for ARV is established using clinical staging and CD4 count (stage I or II with CD4 count < 350 or Stage IV irrespective of CD4 for adults; CD4 < 25% for children less than 11 months and CD4 less than 20% for children between the ages of 12-35 months) as per the Nigerian national guidelines [25]. Drugs, laboratory testing and clinical consultation are provided free of charge.

There are no known studies to the best of our knowledge that provides reliable information on the adverse drug reactions to ART in Nigeria. This study is the largest cohort study conducted in Nigeria and one of the largest cohort studies in West Africa and thus presents complementary information on ADRs in ART care and treatment. The purpose of this paper was to estimate the incidence of known ADRs and to determine risk factors associated with ADRs among HIV positive patients on ARVs.

## Methods

This study was a retrospective cohort analysis of prescription events that were routinely monitored for all patients on ART at the study sites.

### Study sites

The study was conducted at three public hospitals - two secondary level hospitals (Maitama District Hospital, Abuja, and Calabar General Hospital, Calabar) and one tertiary level hospital (Federal Medical Center, Owo). Collectively, they serve over 3000 HIV/AIDS patients with support from the Global HIV/AIDS Initiative, Nigeria (GHAIN), a USAID funded program managed by Family Health International (FHI). Like most public hospitals, the three sites serve mostly uninsured population and operate in an environment characterized by low staff morale, shortage of staff, irregular supply of commodities and weak management systems in general. GHAIN's support to the sites included infrastructure renovations in the clinical area, laboratory and pharmacy, laboratory equipment, training on HIV related topics, provision of job aids, supply of drugs and strengthening the M&E system. An electronic medical record system, the Lafiya Management Information System (LAMIS) was established in these sites since 2007.

### Study population and sample

The cohort included all patients who were initiated on ART between May 2006 and May 2009 and had at least one follow up clinical visit after commencing ARVs between May 2009 and May 2010. Once eligible for ARVs, all patients are initiated on combination antiretroviral therapy consisting of a nucleoside backbone of zidovudine (AZT)/lamivudine (3TC), stavudine (d4T)/3TC, or tenofovir (TDF)/3TC in combination with either nevirapine (NVP) or efavirenz (EFV). Any ARV regimen outside these groups was classified as others. Thereafter, the patient was reviewed monthly for two months. At each appointment, adherence counselling was provided. The patient was subsequently given two monthly prescriptions if found tolerant and adherent to the medication. Baseline CD4, haematology and chemistry test are conducted for all patients and follow up laboratory test scheduled at 3 months and 6 monthly or as determined by the physician. Ethical approval was obtained from the National Health Research Ethics Committee, Nigeria.

### Data collection and management

Active ADR screening commenced in May 2009 under the GHAIN project, however passive screening of ADR had been ongoing and data captured on the patient's ART care card was designated as either yes/no, though details of the ADR was not captured. GHAIN developed a structured ADR screening form modified from World Health Organization and closely related to the ADR form used by the National Food and Drug Agency (NAFDAC). The ADR screening form was designed to identify 38 ADRs that occurred in different organ systems, namely skin and appendages, musculoskeletal system, cardiovascular/respiratory system, central and peripheral nervous system, gastrointestinal/hepato-biliary/renal system, metabolic/endocrine system and systemic signs/symptoms. It also allowed for grading of the ADR reported and documents any intervention provided. ADRs were graded on a four point scale using the W.H.O. severity grading [26]; Grade 1 was classified as "mild" and no limitation of daily activities; Grade 2 classified as "moderate" with mild to moderate limitation of activities; Grade 3 classified as "severe" with marked limitation of activities and Grade 4 classified as "life threatening" with extreme limitation of activities and significant medical intervention.

Clinicians and pharmacist were trained on the content and use of the form by the Medical Services Department of FHI in collaboration with Howard University Continuation Education project (HUCE PACE). They were required to use the form on all patients on ARV at every clinical visit. Each visit screened for an ADR is captured as a yes/no (i.e. yes if an ADR is reported) and binary outcome of 1 was designated if the screening yielded an ADR (i.e. value of 0 if no ADR was reported). All ADRs reported were reviewed by a pharmacovigilance committee (PVC) in each of the sites. Each site had its own PVC which is made up of physicians, pharmacists and laboratory scientists. This committee was responsible for establishing causality of any ADR reported. LAMIS generated an ADR report which provided details of current ART regimen, concomitant medication taken by the patient and most current laboratory parameters of each patient. This information was evaluated per case and causality established before the ADR report is sent to the National pharmacovigilance center under the jurisdiction of the National Agency for Food and Drug Administration and Control. The LAMIS also captured sociodemographic information of each patient such as age, sex and physical address including local government area. All patients clinical encounter (clinical visit, drug refill, laboratory tests) including ADRs were entered into the LAMIS database daily. Data from the LAMIS is backed up daily and domiciled at a central server in a secure location within the hospital. Every month, the data were reviewed by the health facility records officer for completeness and issues identified are discussed at the monthly Multi-LAMIS Evaluation Group (MLEG) meeting. This group was a facility based health management group that worked as a program support group and aimed to ensure program and data ownership and increased data use at the facility level. Members included all cadres of health staff; clinician, pharmacist, nurses, lab scientist, record officers e.t.c.

### Toxicity definition

We used the WHO definition of ADR as any response to a medicine which is noxious and unintended, and which occurs at doses normally used in man [27]. ADRs were evaluated using standard clinical signs and symptoms.

### Data availability

Each LAMIS supported site had three full time data clerks that supported data entry at multiple service delivery areas; general clinic, pharmacy, laboratory and tuberculosis clinic. Patients' data were captured daily and when back log of data occurred, they were captured by the end of the week.

### Statistical analysis

Data from the LAMIS was exported to STATA 10 software (Stata Corporation, College Station, Texas). Follow up data were censored as of May 31^st ^2010. Descriptive and univariate analysis were performed on quantitative data. Median values were used to group CD4 count. Other risk factors include W.H.O. clinical stage, age, sex, duration on treatment and type of regimen prescribed. The Wilcoxon sign rank test was used to compare changes in continuous variables while chi square (*χ*^2^) test was used to test the statistical significance of categorical variables by regimen group. Given the multiple screening of ADR per person, generalized estimating equation (GEE) with logit link was used to determine risk factors associated with ADRs [28,29]. Independence correlation structure was used to account for repeated observations from ADR screening on the same patient over time.

The incidence rate of ADR was expressed as the number of patients with at least one occurrence of the given event per 100 person years [10]. Incidence rate was calculated by time to event method. Patients experiencing an ADR drug reaction were censored at the first occurrence of an ADR. Patients who died, stopped treatment or transferred out to another treatment facility were also censored at time of event. Patients not experiencing any ADR were censored at the end of the observation period. Total time of observation contributed by each patient was summed up to obtain total person years of observation. A *p*-value < 0.05 was considered to be significant for all test conducted.

## Results

### Baseline characteristics

**Figure 1:**
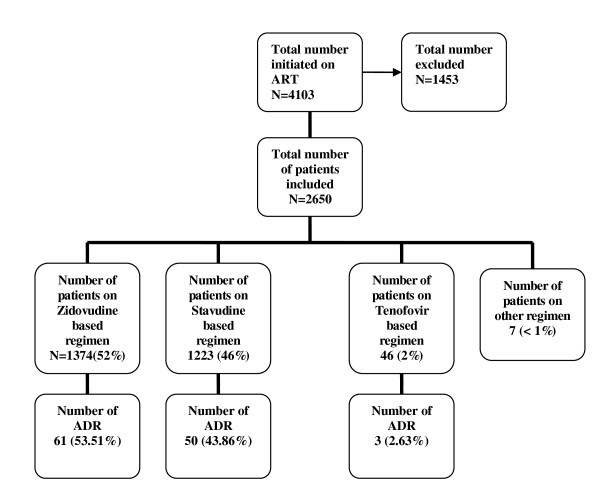
**Distribution of patients by regimen and ADR**.

4103 patients initiated ART between May 2006 and May 2009. 1453 were excluded from the analysis (Figure 1) because they had no clinical visits in the observation period. During the one year of observation period 2,650 patients had 13,479 clinical visits, an average of five visits per patient. Table 1 shows the baseline characteristics of the study population. There were more females 1706 (64%) than males 944 (36%).

**Table 1:** Baseline Characteristics of Study Population

Variable	% (n)
**Sex**	

Female	64.38 (1,706)

Male	35.62 (944)

**Age (years)**	

< 15	6.26 (144)

16-45	80.0 (2120)

> 45	13.74 (364)

**Baseline CD4**	

< = 200	58.72 (1556)

200 - 350	26.68 (707)

> 350	14.60 (387)

Median baseline CD4 (IQR)	172 (85-275)

Baseline WHO Staging*	

Stage I	27.36 (725)

Stage II	26.60 (705)

Stage III	40.23 (1066)

Stage IV	4.08 (108)

**ART Regimen**	

**NRTI Backbone**	

Zidovudine based	51.85 (1374)

Stavudine based	46.15 (1223)

Tenofovir based	1.74 (46)

Others	0.26 (7)

**NNRTI Backbone**	

Nevirapine_based	80.04 (2121)

Efavirenze_based	19.96 (529)

* Total do not add up to 114 due to missing data

The median interval between follow up visits was 2 months (IQR 1.8-2.7). About 80% of the patients were between the ages of 15-46 years, median age was 32 years (IQR 27-40). Clinically, 725 (28%) patients were diagnosed with Stage I disease at initiation. 2139 (81%) patients had at least one CD4 count done during the period under observation. The median baseline CD4 count was 172 cells/μL (IQR 85 - 275). It doubled during follow up, to a median CD4 count of 354 cells/μL (IQR 223 - 517) [p < 0.001].

Of the 2650 patients, 1374 (52%) were on zidovudine based regimen, 1223 (46%) on stavudine based regimen, 46 (1.7%) on tenofovir based regimen and 7 (< 1%) on other regimen. Of the 13, 479 clinical visits, 10,084 (75%) were screened for an ADR and 114 (1.13%) visits reported an ADR.

### Distribution of ADRs

Table 2 shows onset of ADR and distribution by specific characteristics. Of the 114 ADRs reported 83 (73%) had their specific detail collected and 15 patients (28%) reported at least 2 ADRs. Forty-six (45%) of the 114 reported ADRs occurred between 12-24 months on treatment. Sixty-one ADRs (54%) occurred with patients on zidovudine based regimen, 50(44%) with patients on stavudine based regimen and 3 (3%) with patients on tenofovir based regimen.

**Table 2:** Onset and Distribution of ADR

Onset of Adverse Drug Reaction From ART Initiation* (n = 102)	
**Onset of ADR**	**n (%)**

0-3 months	8 (7.84)

3-6 months	14 (13.73)

6-12 months	22 (21.57)

12-24 months	46 (45.10)

24-36 months	10 (9.80)

> 36 months	2 (1.96)

**ADR distribution by age (n = 114)**	

< 15 years	0

16-45 years	97 (85.09)

> 45 years	17 (14.91)

**ADR distribution by gender (n = 114)**	

Male	41 (35.96)

Female	73 (64.04)

* Total do not add up to 114 due to missing data

The ADR screening program implemented by the GHAIN project routinely screened 38 adverse drug reactions and of these 18 (50%) were reported. For severity of ADRs, most of the ADRs reported were Grade 1 (39%), Grade 2 and 3 ADRs were 32% and 28% respectively, while 1% of ADRs was found to be life threatening. Amongst those that reported an ADR, no change in regimen was documented.

### Incidence of ADRs

Patients were followed up for a total of 2456 person-years yielding an incidence rate (IR) of 4.6/100 person-years. Overall incidence of ADR by nucleoside backbone was 4.4%, 4.1% and 6.5% for patients on AZT, d4T and TDF based regimen respectively while by non-nucleoside base, incidence was 4.2% and 4.9% for patients on nevirapine and efavirenz respectively.

Table 3 shows the incidence rates of reported ADR by regimen group. Pain/tingling/numbness was the most common ADR reported (30%) with an IR of 2.1 and 0.1 per 100 person-years among patients on d4T and AZT based regimen respectively, followed by skin rash (18%) with an IR of 2.6, 0.5 and 0.6 per 100 person years among patients on TDF, d4T and AZT respectively.

**Table 3:** Incidence Rates of ADR by Regimen per 100 person-years of Treatment

Specific ADR by Organ System	AZT_based (n)	d4T_based (n)	TDF_based (n)	Total (%)
**Gastrointestinal**				

Abdominal pain	-	0.09 (1)	-	1 (1.20)

Diarrhea	0.32 (4)	-	-	4 (4.82)

Dyspepsia	0.08 (1)	-	-	1 (1.20)

Nausea & Vomiting	0.24 (3)	-	-	3 (3.61)

**Cardiovascular/Respiratory System**				

Chest pain	-	0.09 (1)	-	1 (1.20)

Cough	-	0.09 (1)	-	1 (1.20)

**Skin and Appendages**				

Pruritus	0.24 (3)	0.61 (7)	-	10 (12.05)

Steven Johnson syndrome	0.08 (1)	-	-	1 (1.20)

Skin rash	0.64 (8)	0.52 (6)	2.56 (1)	15 (18.07)

**Central and Peripheral Nervous System**				

Headache	0.08 (1)	0.09 (1)	-	2 (2.41)

Dizziness	0.08 (1)	0.44 (5)	-	6 (7.23)

Insomnia	0.16 (2)	-	-	2 (2.41)

Nightmare	0.08 (1)	0.09 (1)	-	2 (2.41)

Pain/Tingling/Numbness	0.08 (1)	2.09 (24)	-	25 (30.12)

**Musculoskeletal System**				

Myalgia	0.08 (1)	-	-	1 (1.20)

**Systemic Signs/Symptoms**				

Rigor	0.08 (1)	-	-	1 (1.20)

Fatigue	0.08 (1)	-	-	1 (1.20)

Fever	0.08 (1)	-	-	1 (1.20)

**Total**	**34**	**48**	**1**	**83**

### Risk factors for ADR

Table 4 shows risk factors associated with ADRs. The adjusted GEE analysis showed that patients on longer duration of treatment had decreased odds of developing an ADR compared to patients in their first six months on treatment, 6-12 months (AOR 0.38, 95% CI 0.16-0.91, *P = *0.03), 12-24 months (AOR 0.35, 95% CI 0.16-0.73, *P *= 0.005), 24-36 months (AOR 0.37, 95% CI 0.14-0.96, *P = *0.05). With TDF based regimen as the reference, patients on d4T (AOR 0.18, CI 0.05-0.64, p = 0.009) and AZT (AOR 0.24, CI 0.07-0.904, p = 0.034) were less likely to report an ADR. Age (AOR 1.27, CI 0.59-2.77, p = 0.544) gender (AOR 0.83, 0.43-1.57, p = 0.562) and CD4 count (AOR 0.93, CI 0.51-1.70, p = 0.816) were not significantly associated with developing an ADR.

**Table 4:** Generalized Estimating Equation Analysis for Risk Factors Associated with ADR

Variables	N (%)	Unadjusted OR	*p*	Adjusted OR	*p*
**Gender**					

Female	1706 (64.4)				

Male	944 (35.6)	1.11(0.71-1.73)	0.663	0.83 (0.43-1.57)	0.562

**Age (years)**					

15-45	2120 (85.3)				

> 45	364 (14.7)	0.81(0.48-1.36)	0.422	1.27 (0.59-2.77)	0.544

**Regimen**					

TDF_based	46 (1.7)				

AZT_based	1374 (52.0)	0.56 (0.18-1.75)	0.319	0.24 (0.07-0.90)	0.034

D4T_based	1223 (46.3)	0.33 (0.10-1.03)	0.057	0.18 (0.05-0.64)	0.009

**Duration on treatment (months)**					

0-6	21 (0.79)				

6-12	79 (2.98)	0.54 (0.33-0.89)	0.02	0.38 (0.16-0.91)	0.03

12-24	259 (47.5)	0.25 (0.15-0.41)	< 0.001	0.35 (0.16-0.73)	0.005

24-36	952 (35.9)	0.16 (0.08-0.34)	< 0.001	0.37 (0.14-0.96)	0.042

**CD4 cell count cells/μL**					

< 350	950 (44.8)				

> 350	1180 (55.2)	1.08 (0.6-2.0)	0.791	0.93 (0.51-1.70)	0.816

## Discussion

The likelihood of developing an adverse drug reaction was highest in the first six months of commencing antiretroviral therapy. Xavier et al. [4] proffered an explanation that early occurrence of ADRs is an expression of a mechanism of intrinsic intolerance rather than of a time-dependent toxic accumulation process. Close monitoring of patients within this time frame is thus imperative to prevent the occurrence of severe ADRs, improve adherence as well as improve documentation of ADRs. However 45% of the reported ADRs occurred within 12-24 months of commencing ARVs. This calls for the need to intensify long term ADR monitoring in patients on ARV. Some studies have proposed time-dependent toxic accumulation as the mechanism of developing an ADR long after commencing medication. Thus monitoring for ADR should be an ongoing process as we have both early onset and late onset ADRs. Adding a laboratory component to the ADR screening would go a long way in determining biochemical markers that would help to improve patient management. However from a programmatic aspect in a resource constrained environment, having sound knowledge of the risk factors or common ADRs associated with different ARV regime can help focus scarce resources to managing ADRs in these settings.

Since adverse drug reactions are the single most common reason for poor adherence to treatment, identifying risk factors for the occurrence of ADRs is of crucial importance to optimize the initial choice of ARVs regimen before initiating therapy and to adapt the pace of surveillance to each unique situation [4]. Our study showed no difference in reported ADR between men and women, however Bonfati et al [7]. observed that women experienced significantly greater number of adverse effects compared to men. Though the population of patients on tenofovir based regimen was small compared to AZT and d4T, our data shows that patients on AZT or d4T were less likely to report an ADR than those on TDF. A multisite trial in Africa, found tenofovir therapy to be associated with 1.3% rate of significant nephrotoxicity which was comparable to other regimen,[5,30] thus showing no significant toxicity difference between tenofovir and other regimens. This raises a sentinel sign that perhaps drug response to TDF in this setting is not in conformity with the results from other studies where drug profile of TDF has been superior over AZT and d4T. A closer look at the drug profile and toxicity of TDF is urgently needed to better understand its tolerance in patients in this setting. Furthermore, the most common side effect of tenofovir is renal impairment as measured by reduced creatinine clearance,[31] thus as tenofovir replaces d4T as the nucleoside backbone of choice in HIV treatment, laboratories in resource poor settings must be strengthened to able to conduct this test.

Incidence of anaemia was low at 4% and occurred exclusively in patients on AZT. This is similar to other studies conducted in Nigeria, Coˆ te d'Ivoire, Haiti and India that observed anaemic rates of 3%-12% [5,32-38]. The incidence of skin toxicity (18%) is similar to that in other reports,[15,17] though some reports have observed low incidence of skin toxicity,[10,31] however the incidence of Steven-Johnson syndrome (1%) was similar to other reports which reported less than 5% [14-17]. Most of the reported ADRs (71%) were mild to moderate and self limiting in nature while 1% were life threatening. This suggests good tolerance level to ARVs in general. While other studies have associated low CD4 count at treatment initiation as a risk factor for ADR [5], our study did not show any association between CD4 cell count and clinical stage with ADRs.

Our study takes strength in its large sample size. This is the largest cohort of patients who have been surveyed in Nigeria for ADR using active surveillance. It also presents ADR outcomes in a large public health program and more closely presents treatment outcomes that are more generalizable than clinical studies. Finally data in this study was of good quality giving the scale of the program and its routine nature of collection. Mathieu Forster et al. [39] assessed data quality for ART services in low income countries by evaluating the availability of six key variables (age, sex, W.H.O clinical staging at baseline and follow-up, CD4 count and year of ART initiation) and calculating the proportion of missing data to determine the quality of data and the median was found to be 10.9%. The median of the percentages of missing variables was 0% for all sites surveyed.

This study has some limitations. The study included patients who had initiated ART before active surveillance of ADR commenced. Though this provided information on long term adverse effects, we may have missed early onset ADR from these patients. The small sample size of patients on tenofovir based regimen limits our ability to compare ADR reported by this group with other regimen groups. Also the ADR screening tool was structured and thus, does not allow details of unknown ADR to be captured and graded, thus the study was confined to report on known ADRs only. Finally, not all ADRs reported had their complete details collected and graded. Thus the specific ADRs in this study are most likely under reported.

## Conclusion

Incidence rate (4.6/100 person years) of ADRs is low amongst patients on ARVs. Active screening of ADRs has increased the documentation of the occurrence of these events and should be scaled up to all facilities providing comprehensive care to HIV patients. Improving quality of care to patients by providing ADR screening provides an avenue for early identification and subsequently treatment of adverse drug reactions. Further evaluation of patients on tenofovir would be beneficial in documenting adverse drug reactions related to tenofovir as well as its tolerability. Finally more field based studies in resource constrained settings should be conducted and ADRs related to ARVs evaluated and compared to ADRs observed from clinical trials. This will provide valuable insight in the incidence, prevalence and type of ADRs associated with ARVs.

## Competing interests

The author declares that they have no competing interests.

## Authors' contributions

GE conceived the study, performed the statistical analysis and drafted the manuscript. KA drafted the manuscript and provided critical review of the article. TB participated in study design, performed statistical analysis and reviewed the article. All authors read and approved the final manuscript

## Pre-publication history

The pre-publication history for this paper can be accessed here:

http://www.biomedcentral.com/1472-6904/12/7/prepub

## References

[B1] Detels R Muñoz A McFarlane G Kingsley LA Margolick JB Giorgi J Schrager LK Phair JP Effectiveness of potent antiretroviral therapy on time to AIDS and death in men with known HIV infection duration AIDS Cohort Study Investigators JAMA 1998 280 1497 1503 10.1001/jama.280.17.1497 9809730

[B2] Hogg RS Yip B Kully C Craib KJ O'Shaughnessy MV Schechter MT Montaner JS Improved survival among HIV infected patients after initiation of triple drug antiretroviral regimens CMAJ 1999 160 Suppl 5 659 665 10102000 PMC1230111

[B3] Palella FJ Jr Delaney KM Moorman AC Loveless MO Fuhrer J Satten GA Aschman DJ Holmberg SD Declining morbidity and mortality among patients with advanced human imunodeficiency virus infection. HIV outpatient Study Investigators N Engl J Med 1998 338 853 860 10.1056/NEJM199803263381301 9516219

[B4] Duval X Journot V Leport C Chêne G Dupon M Cuzin L May T Morlat P Waldner A Salamon R Raffi F Antiprotease Cohort (APROCO) Study Group Incidence of and risk factors for adverse drug reactions in a prospective cohort of HIV infected adults initiating protease inhibitor containing therapy Infectious Dis Soc Am 2004 39 248 255 10.1086/422141 15307035

[B5] Subbaraman R Chaguturu SK Mayer KH Flanigan TP Kumarasamy N Adverse effects of highly active antiretroviral therapy in developing countries Clin Infectious Dis 2007 45 1093 1101 10.1086/521150 17879931

[B6] Patrice S Juste MA Ambroise A Eliacin L Early versus standard antiretroviral therapy for HIV infected adults in Haiti N Engl J Med 2010 363 257 265 10.1056/NEJMoa0910370 20647201 PMC3676927

[B7] Bonfanti P Valsecchi L Parazzini F Carradori S Pusterla L Incidence of adverse reactions in HIV patients treated with protease inhibitors: a cohort study JAIDS 2000 23 236 245 10.1097/00126334-200003010-00004 10839659

[B8] Dean GL Edwards SG Ives NJ Matthews G Fox EF Navaratne L Fisher M Taylor GP Miller R Taylor CB de Ruiter A Pozniak AL Treatment of tuberculosis in HIV infected persons in the era of highly active antiretroviral therapy AIDS 2002 16 75 83 10.1097/00002030-200201040-00010 11741165

[B9] World Health Organization Pharmacovigilance for antiretrovirals in resource poor countries. medicines policy and standards Geneva, World Health Organization 2007

[B10] Laurent C Bourgeois A Mpoudi-Ngolé E Ciaffi L Kouanfack C Mougnutou R Nkoué N Calmy A Koulla-Shiro S Delaporte E Tolerability and effectiveness of first line regimens combining nevirapine and lamivudine plus zidovudine or stavudine in Cameroon AIDS Res Human Retroviruses 2008 24 Suppl 3 393 399 10.1089/aid.2007.0219 18327976

[B11] Pollard RB Robinson P Dransfield K Safety profile of nevirapine, a nonnucleoside reverse transcriptase inhibitor for the treatment of human immunodeficiency virus infection Clin Ther 1998 20 1071 1092 10.1016/S0149-2918(98)80105-7 9916603

[B12] (DHHS), U. S. D. o. H. a. H. S Guidelines for the use of antiretroviral agents in HIV-1 infected adults and adolescents Panel on antiretroviral guidelines for adults and adolescents 2006

[B13] Gazzard B Bernard AJ Boffito M Churchill D Edwards S Fisher N Geretti AM Johnson M Leen C Peters B Pozniak A Ross J Walsh J Wilkins E Youle M British HIV association (BHIVA) guidelines for the treatment of HIV infected adults with antiretroviral therapy HIV Med 2006 7 Suppl 8 487 503 17105508 10.1111/j.1468-1293.2006.00424.x

[B14] Colebunders R Kamya MR Laurence J Kambugu A Byakwaga H Songa Mwebaze P Muganzi Muganga A Katwere M Katabira E Muganzi A First-line antiretroviral therapy in Africa, how evidence based are our recommendations? AIDS Rev 2005 7 Suppl 3 148 154 16302462

[B15] Montessori V Press N Harris M Akagi L Montaner JSG Adverse effects of antiretroviral therapy for HIV infection Can Med Assoc Licensors 2004 170 Suppl 2 229 238 PMC315530 14734438

[B16] Jamisse L Balkus J Hitti J Gloyd S Manuel R Osman N Djedje M Farquhar C Antiretroviral associated toxicity among HIV 1 seropositive pregnant women in Mozambique receiving nevirapine based regimens J Acquir Immune Defic Syndr 2007 44 Suppl 4 371 376 17259905 10.1097/QAI.0b013e318032bbee

[B17] Phanuphak N Apornpong T Teeratakulpisarn S Chaithongwongwatthana S Taweepolcharoen C Mangclaviraj S Limpongsanurak S Jadwattanakul T Eiamapichart P Luesomboon W Apisarnthanarak A Kamudhamas A Tangsathapornpong A Vitavasiri C Singhakowinta N Attakornwattana V Kriengsinyot R Methajittiphun P Chunloy K Preetiyathorn W Aumchantr T Toro P Abrams EJ El-Sadr W Phanuphak P Nevirapine associated toxicity in HIV-infected Thai men and women, including pregnant women Br HIV Assoc 2007 8 357 366 10.1111/j.1468-1293.2007.00477.x 17661843

[B18] Dieterich DT Love J Stern JO Drug-induced liver injury associated with the use of nonnucleoside reverse-transcriptase inhibitors Clin Infect Dis 2004 38 Suppl 2 S80 S89 14986279 10.1086/381450

[B19] Baylor MS Johann-Liang R Hepatotoxicity associated with nevirapine use J Acquir Immune Defic Syndr 2004 35 538 10.1097/00126334-200404150-00014 15021321

[B20] Stern JO Robinson PA Love J Lanes S Imperiale MS Mayers DL A comprehensive hepatic safety analysis of nevirapine in different populations of HIV-infected patients J Acquir Immune Defic Syndr 2003 34 S21 S33 10.1097/00126334-200309011-00005 14562855

[B21] de Maat MM ter Heine R van Gorp EC Mulder JW Mairuhu AT Beijnen JH Case series of acute hepatitis in a non-selected group of HIV-infected patients on nevirapine containing antiretroviral treatment AIDS 2003 17 2209 2214 10.1097/00002030-200310170-00009 14523278

[B22] Martínez E Blanco JL Arnaiz JA Pérez-Cuevas JB Mocroft A Cruceta A Marcos MA Milinkovic A García-Viejo MA Mallolas J Carné X Phillips A Gatell JM Hepatotoxicity in HIV infected patients recieving nevirapine containing antiretroviral therapy AIDS 2001 15 1261 1268 10.1097/00002030-200107060-00007 11426070

[B23] Sulkowski MS Thomas DL Mehta SH Chaisson RE Moore RD Hepatotoxicity associated with nevirapine or efavirenz containing antiretroviral therapy:role of hepatitis C and B infections Hepatology 2003 1 35 10.1053/jhep.2002.30319 11786975

[B24] Asuzu MC The necessity for a health systems reform in Nigeria J Community Med & Primary Health Care 2000 16 1 322353190

[B25] Federal Ministry of Health Abuja, Nigeria National guidelines for HIV/AIDS treatment and care in adolescents and adults 2010

[B26] World Health Organization Antiretroviral therapy for HIV infection in adults and adolescents: recommendations for a public health approach WHO 2006 28 http://www.who.int/hiv/pub/guidelines/artadultguidelines.pdf (accessed December 3rd, 2011)

[B27] World Health Organization Assuring safety of preventive chemotherapy interventions for neglected tropical diseases. practical advice for national programme managers on the prevention, detection and management of serious adverse effects WHO 2011 1 http://whqlibdoc.who.int/publications/2011/9789241502191_eng.pdf (accessed December 3^rd^, 2011)

[B28] Liang KY Zeger SL Longitudinal data analysis using generalized linear models Biometrica 1986 73 13 22 10.1093/biomet/73.1.13

[B29] Liang KY Zeger SL Regression analysis for correlated data Annu Rev Publ Health 1993 14 43 68 10.1146/annurev.pu.14.050193.000355 8323597

[B30] Reid A Walker S Ssali F Munderi P Gilks C Glomerular dysfunction and associated risk factors following initiation of ART in adults with HIV infection in Africa [abstract THAB0105] International AIDS Society 2006

[B31] Forna F Liechty CA Solberg P Asiimwe F Were W Mermin J Behumbiize P Tong T Brooks JT Weidle PJ Clinical toxicity of highly active antiretroviral therapy in a home-based AIDS care program in rural Uganda J Acquir Immune Defic Syndr 2007 44 Suppl 4 456 462 17279048 10.1097/QAI.0b013e318033ffa1

[B32] Kumarasamy N Lai A Cecelia AJ Toxicities and adverse events following generic HAART in south Indian HIV-infected individuals [abstract P189] Proceedings of the 7th International Congress on Drug Therapy and HIV Infection (Glasgow, United Kingdom) 2004

[B33] Idoko JA Akinsete L Abalaka AD Keshinro LB Dutse L Onyenekwe B Lhekwaba A Njoku OS Kehinde MO Wambebe CO A multicentre study to determine the efficacy and tolerability of a combination of nelfinavir (VIRACEPT), zalcitabine (HIVID) and zidovudine in the treatment of HIV infected Nigerian patients West Afr J Med 2002 21 83 86 12403023

[B34] Moh R Danel C Sorho S Sauvageot D Anzian A Minga A Gomis OB Konga C Inwoley A Gabillard D Bissagnene E Salamon R Anglaret X Haematological changes in adults receiving a zidovudine-containing HAART regimen in combination with cotrimoxazole in Coˆ te d'Ivoire Antivir Ther 2005 10 615 624 16152755 10.1177/135965350501000510

[B35] Shah I Adverse effects of antiretroviral therapy in HIV-1 infected children J Trop Pediatr 2006 52 244 248 16126803 10.1093/tropej/fmi086

[B36] Severe P Leger P Charles M Noel F Bonhomme G Bois G George E Kenel-Pierre S Wright PF Gulick R Johnson WD Jr Pape JW Fitzgerald DW Antiretroviral therapy in a thousand patients with AIDS in Haiti N Engl J Med 2005 353 2325 2334 10.1056/NEJMoa051908 16319381

[B37] Bygrave H Kranzer K Hilderbrand K Jouquet G Goemaere E Vlahakis N Triviño L Makakole L Ford N Renal safety of tenofovir containing first line regimen: experience from an antiretroviral cohort in rural Lesotho PLoS One 2011 6 Suppl 3 e17609 21407815 10.1371/journal.pone.0017609 PMC3047584

[B38] Lucas GM Moore RD Highly active antiretroviral therapy in a large urban clinic: risk factors for virologic failure and adverse drug reactions Ann Intern Med 1999 131 Suppl 2 81 87 10419445 10.7326/0003-4819-131-2-199907200-00002

[B39] Forster M Bailey Christopher Brinkhof Martin WG Electronic medical record systems, data quality and loss to follow-up: survey of antiretroviral therapy programs in resource-limited settings Bull World Health Organization 2008 86 939 947 10.2471/BLT.07.049908 PMC2649575 19142294

