# Correction: Adverse drug reactions to antiretroviral therapy (ARVs): incidence, type and risk factors in Nigeria

**DOI:** 10.1186/1472-6904-12-14

**Published:** 2012-06-28

**Authors:** George I Eluwa, Titilope Badru, Kenneth A Agu, Kesiena J Akpoigbe, Otto Chabikuli, Christoph Hamelmann

**Affiliations:** 1 Department of Operations Research, HIV/AIDS Program. Population Council, Nigeria. No. 16, Mafemi Crescent, Utako, Abuja, Nigeria; 2 Department of Health Policy and Management, Diadem Consults Ltd, Abuja, Nigeria; 3 Management Sciences for Health, Abuja, Nigeria; 4 Howard University PACE Center, Washington, USA; 5 Society for Family Health, Abuja, Nigeria; 6 Family Health International 360, Pretoria, South Africa; 7 UNDP Regional Center for Europe and Central Asia, Bratislavia, Slovakia

## Correction

After publication of the article it was noted that we inadvertently failed to include the complete list of authors. The full list, including co-authors, has now been added and the Authors' contributions section modified accordingly.

## **Authors’ Contributions**

GIE conceived the study. GIE, CH, KAA and OC contributed to the study design and implementation. GIE, TB, OC and KJA performed the statistical analysis. GIE, TB, OC, KAA, KJA and CH participated in data interpretation. GIE, KJA, KAA, OC, TB and CH drafted the manuscript. TB, KJA, KAA, OC and CH provided critical review of the article. All authors read and approved the final manuscript.

## Pre-publication history

The pre-publication history for this paper can be accessed here:

http://www.biomedcentral.com/1472-6904/12/14/prepub
